# The role of vestibular function tests in nontuberculous mycobacterial otomastoiditis: A case report

**DOI:** 10.1097/MD.0000000000037007

**Published:** 2023-02-02

**Authors:** Hann-Ziong Yueh, Hung-Lun Chu, Shih-Chun Lu, Yuarn-Jang Lee, Che-Hsuan Lin

**Affiliations:** a Department of Otolaryngology, Taipei Medical University Hospital, Taipei, Taiwan; b Department of General Medicine, Taipei Medical University Hospital, Taipei, Taiwan; c Division of Infectious Diseases, Department of Internal Medicine, Shuang Ho Hospital, Taipei Medical University, New Taipei City, Taiwan; d Division of Infectious Diseases, Department of Internal Medicine, School of Medicine, College of Medicine, Taipei Medical University, Taipei, Taiwan; e Department of Otolaryngology, School of Medicine, College of Medicine, Taipei Medical University, Taipei, Taiwan.

**Keywords:** case report, dizziness, nontuberculous mycobacteria, otomastoiditis, vestibular tests

## Abstract

**Background:**

Nontuberculous mycobacteria (NTM), an extremely rare pathogen causing cervicofacial infections, may result in permanent hearing impairment or intracranial complications. Due to the lack of specific manifestations during the initial onset of NTM otomastoiditis, physicians may misdiagnose it as cholesteatoma or other common bacterial infections.

**Patient concerns:**

A 44-year-old male who complained of left-sided aural fullness, otalgia, and dizziness for 2 months.

**Diagnosis:**

The initial diagnosis was hypothesized to be cholesteatoma based on a whitish mass with mucoid discharge filling the entire outer ear canal on otoscopy and left-sided mixed hearing loss. However, NTM was identified by microbial culture at the 2-month follow-up after surgery.

**Interventions:**

The patient underwent a left-sided exploratory tympanotomy. Because NTM otomastoiditis was diagnosed, 3 weeks of starting therapies were administered with azithromycin (500 mg/day, oral administration), cefoxitin (3 g/day, intravenous drip), and amikacin (750 mg/day, intravenous drip). The maintenance therapies were azithromycin (500 mg/day, oral administration) and doxycycline (200 mg/day, oral administration) for 7 months.

**Outcomes:**

The patient’s clinical condition improved initially after surgery, but the otomastoiditis gradually worsened, combined with subtle meningitis, 2 months after surgery. The external auditory canal became swollen and obstructed, making it difficult to monitor the treatment efficacy through otoscopy. Thus, we used regular vestibular function tests, including static posturography, cervical vestibular evoked myogenic potentials, and video Head Impulse Test, to assess recovery outcomes. After antibiotic treatment, the infectious symptoms subsided significantly, and there was no evidence of infection recurrence 7 months after treatment. Improvements in static posturography and cervical vestibular evoked myogenic potentials were compatible with the clinical manifestations, but video Head Impulse Test showed an unremarkable correlation.

**Lessons:**

The clinical condition of NTM otomastoiditis may be evaluated using vestibular tests if patients have symptoms of dizziness.

## 1. Introduction

The most common cervicofacial infections in ENT include cervicofacial lymphadenitis, tonsillitis, and salivary gland infections. Otomastoiditis is rarely reported in patients with cervicofacial infections. The most common pathogens associated with otomastoiditis are *Streptococcus pneumoniae* and *Haemophilus influenzae*, whereas *Mycobacterium tuberculosis* and nontuberculous mycobacteria (NTM) are relatively rare.^[1]^ NTM, which are found ubiquitously in soil and water, are well-known to cause a broad spectrum of diseases, including lymphadenitis, pulmonary disease, ulcerative skin lesions, injection abscesses, and disseminated infections, especially in immunodeficient patients.^[2]^ NTM otomastoiditis may present with chronic otorrhea and exuberant middle ear granulation tissue, which tends to cause long-standing infections that can involve the meninges and brain.^[3]^ Approximately 17% of otomastoiditis patients experience concurrent intracranial complications.^[4]^

Several species have been described as being responsible for NTM otomastoiditis, such as *Mycobacterium abscessus, M. chelonae, M. fortuitum*, and *M. avium* complex. *M. abscessus* is the most common NTM and has a tendency to cause mastoiditis.^[5]^ In the pediatric population, *M. abscessus* has been reported to be the most frequently isolated organism in otitis media and otomastoiditis caused by NTM.^[6]^ It is susceptible to clarithromycin, azithromycin, and aminoglycoside amikacin. In addition to prolonged antibiotic treatment, the majority of previously reported cases required surgery, and in many cases, mastoidectomy was performed.^[3]^ Herein, we present a rare case of NTM otomastoiditis presenting with aural fullness, otalgia, and dizziness. Clinical evaluations of vestibular tests were continuously performed at the outpatient clinic for outcome assessment. The patient was successfully treated with antibiotic therapy without other medications.

## 2. Case report

**Figure 1.**
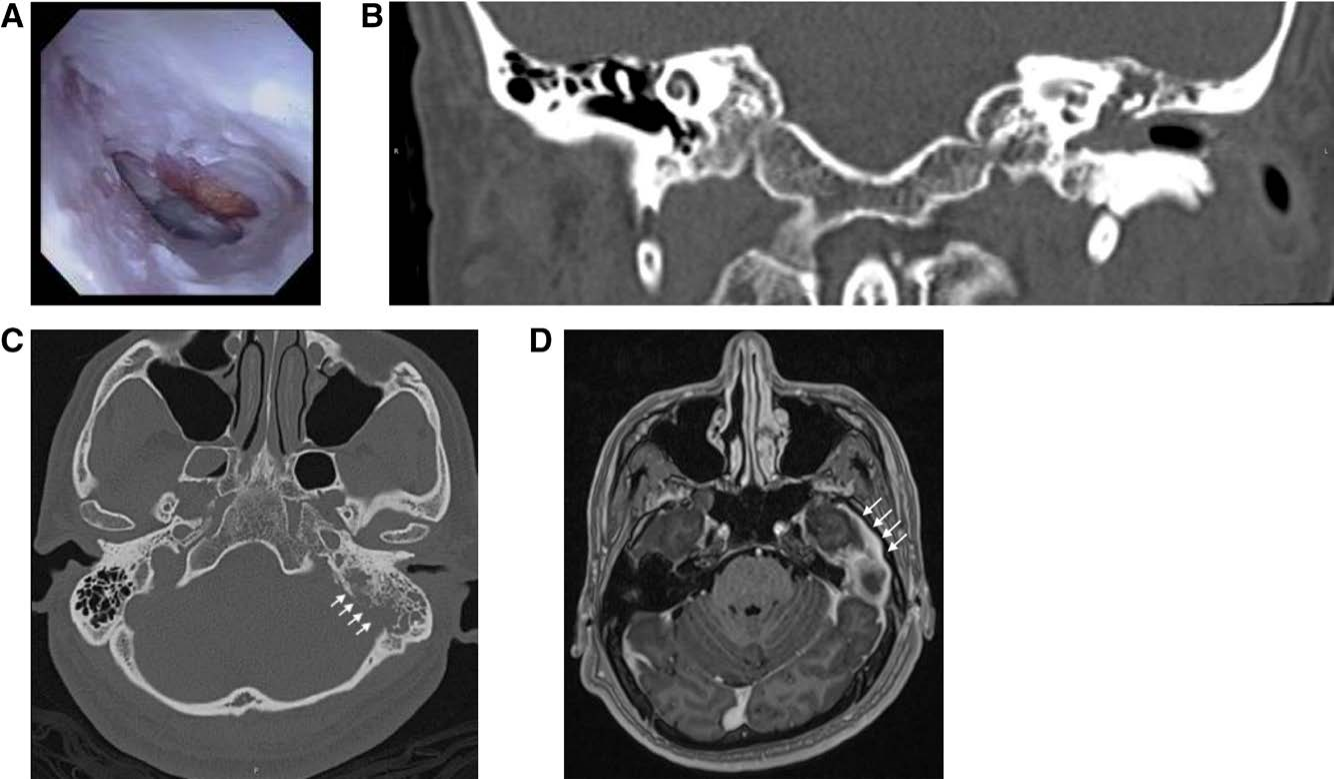
(A) Otoscopic preoperative evaluation, granulations at the attic, and posterosuperior quadrant. (B,C) CT temporal bone, (B) axial and (C) coronal view, showing initial left cavity and soft tissue lesion, and recurrent erosions of mastoid (white solid arrow). (D) MRI showed left otomastoditis with subtle meningitis. CT = computed tomography, MRI = magnetic resonance imaging.

A 44-year-old man with no history of otologic disease presented to our ENT outpatient clinic with a 2-month history of left-sided aural fullness, otalgia, and dizziness. Topical antibiotic ear drops were prescribed by local hospitals, but his condition responded poorly to treatment. Otoscopy revealed a whitish lesion located in the posterior quadrant of the left middle ear with an intact tympanic membrane, which was initially suspected to be a cholesteatoma (Fig. 1A), and the entire external canal was swollen and filled with mucoid discharge. Pure-tone audiometry (PTA) showed mixed hearing loss (A-B Gap, 33.75 dB) on the left side. Tympanometry revealed a type As tympanogram on the left side and type Ad on the right side. Computed tomography (CT) of the temporal bone demonstrated soft tissue density in the left middle ear canal with erosion of the ossicles, a suspected chronic otitis media associated with cholesteatoma (Fig. 1B,C). In view of frequent symptoms, progressive bony destruction on follow-up CT scans, and the refractory nature of the patient’s disease with conservative treatment, he underwent left-sided exploratory tympanotomy for disease control. During surgery, there was no definite cholesteatoma in the middle ear after perioperative elevation of the tympanic membrane; instead, plenty of granulations with chronic inflammation were found. Postoperative pathology revealed an inflamed granulation tissue with lymphocytic infiltration. Three weeks after surgery, otoscopy revealed a grossly intact left eardrum with some discharge. However, at the 2-month follow-up after the surgery, the patient’s otorrhea gradually worsened, and the external auditory canal was so swollen that the eardrum could not be evaluated. In addition, the patient progressively developed aural fullness, headache, dizziness, and inability to maintain balance. Under the impression of postoperative infection, ciprofloxacin was administered based on the bacterial culture result of *Pseudomonas aeruginosa*, which showed little improvement. Due to deteriorating clinical conditions, brain magnetic resonance imaging (MRI) and temporal bone CT were performed for further imaging studies. Brain MRI revealed left chronic coalescent otomastoditis associated with subtle meningitis (Fig. 1D), and temporal bone CT showed left external and middle ear cavities, mastoid antrum isodense debris, left inferior-inner mastoid antrum cortical destruction, and suspected chronic coalescent otomastoditis or other superimposed infection/inflammatory processes. The patient was admitted to our ward and received empirical intravenous antibiotic therapy with amoxicillin/clavulanic acid. Unexpectedly, microbial culture identified the presence of NTM, and DNA sequence amplification by polymerase chain reaction identified the isolated microorganism as *M. abscessus*. Therefore, depending on the culture report, azithromycin (500 mg/d, oral administration), cefoxitin (3 g/d, intravenous drip), and amikacin (750 mg/d, intravenous drip) were subsequently administered. Within 3 weeks of starting therapy, maintenance antibiotic therapy with azithromycin (500 mg/d, oral administration) and doxycycline (200 mg/d, oral administration) were administered for 7 months. The symptoms subsided significantly in the ear and general condition, and remained well without evidence of infection recurrence 7 months after treatment. Rechecked otoscopy showed a well-epithelialized cavity without residual granulations/polypoid tissue or discharge.

**Figure 2.**
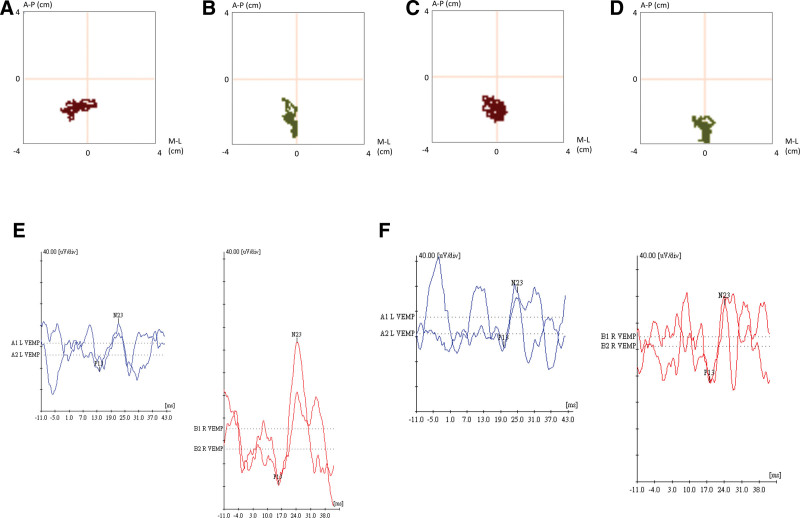
Followed up of vestibular tests about sPSG and cVEMP. Static posturography expressed by the center of pressure position on the horizontally placed XY plane, anteroposterior (A-P) and mediolateral (M-L) direction. (A) Eyes open and (B) eyes closed in the first examination, (C) eyes open and (D) eyes closed in the examination after half year treatments. (E) cVEMP amplitude asymmetry with 47% at the first examination. (F) Amplitude asymmetry with 14% followed after half year treatments. cVEMP = cervical vestibular evoked myogenic potentials, sPSG = static posturography.

Static posturography (sPSG), cervical vestibular evoked myogenic potentials (cVEMP), and video head impulse test (vHIT) have been regularly followed up for evaluation of treatment outcome, since we found that his external auditory canal was too swollen to be evaluated by the otoscope. Interestingly, sPSG and cVEMP showed obvious improvement compared with the initial results and were compatible with clinical manifestations, but vHIT showed an unremarkable correlation. sPSG indicated that the sizes of stabilogram surface areas of 123.6 mm^2^ in eyes open and 509.0 mm^2^ in eyes closed at the first evaluation, and 88.9 mm^2^ in eyes open and 151.8 mm^2^ in eyes closed in half a year followed. cVEMPs were evoked by air-conducted sounds of 95 dB nH and 500 Hz tone bursts. The stimulus was present on the right ear but attenuated on the left ear during the first cVEMPs examination with 47% amplitude asymmetry. The left cVEMPs improved significantly with 14% amplitude asymmetry after half a year (Fig. 2). Unfortunately, this patient did not show improvement in the PTA test, indicating that NTM otomastoiditis caused permanent hearing impairment.

## 3. Discussion

Diagnosing otomastoiditis caused by NTM infection is challenging because the presentation of a variety of nonspecific signs and symptoms makes it a diagnostic dilemma. The clinical presentations are described as a painless, chronically draining ear that does not respond to antibacterials, pale granulations in the aural canal, severe sensorineural hearing loss, or retroauricular swelling, which result from its mass, bony destruction, and secondary infection.^[3,5,7]^ Severe hearing loss most often occurs early on and can be sensorineural, conductive, or mixed.^[8]^ However, dizziness is rarely mentioned or emphasized in previous literature. Otoscopy may detect polyps and granulations over the external auditory canal, middle ear cavity, or mastoid area, similar to that observed in chronic otitis media caused by common pathogens. Occasionally, physicians may mistakenly identify NTM otomastoiditis as cholesteatoma because both of these diseases result in erosion of the ossicles in the middle ear and consequent hearing loss,^[9,10]^ even invading directly into the inner ear and causing severe dizziness, as shown in our case.

In knowledge of the radiological findings, CT of the temporal bone cannot differentiate NTM from other forms of otitis media or granulomatous disease but is generally performed to rule out bone erosions and intracranial complications.^[11]^ Furthermore, MRI can provide new additional information regarding tissue relaxation times of chronic inflammatory soft tissue deposition.^[12]^ Saat et al^[13]^ reported that MRI was inferior to CT in direct estimation of bone defects, but superior to CT in detecting intracranial infection. In a previous case series, ossicular erosions tended to be seen in tuberculous mastoiditis and chronic otomastoiditis with cholesteatoma.^[10]^ Therefore, proper assessment of bone and soft tissue changes is still a challenge for physicians to initiate medical treatment to eradicate the infection and determine whether surgical intervention is necessary to prevent potential complications.

Unlike tuberculosis, the sensitivity of NTM to current antibiotic agents is variable and often limited.^[7]^ Treatment with a single drug usually leads to resistance and clinical failure, making the infection difficult to control. Therefore, the ideal treatment for NTM otomastoiditis usually requires a combination of surgery and antibiotics. The American Thoracic Society guidelines for the treatment of soft tissue and bone infections caused by *M. abscessus* advocate 4 to 6 months of therapy with a macrolide, aminoglycoside, and cefoxitin or carbapenem, based on in vitro drug susceptibility test results, combined with surgical debridement when possible.^[2]^ In this case, the use of a short period of intravenous antibiotics and continued oral antibiotic therapy.

During the therapeutic course, it is difficult to evaluate the internal condition of the ear when the external auditory canal is blocked by swelling granules. Therefore, it is crucial to seek an alternative approach to effectively monitor the progression of the condition when a swollen external auditory canal obstructs the observation of the middle ear through an otoscope. It is not aligned with the cost-effectiveness principles that we perform CT or MRI scans to follow the condition of bone erosion, soft tissue inflammation, and intracranial infection during each outpatient follow-up. Moreover, since otomastoiditis may result in permanent hearing impairment, PTA, or other audiometry tests cannot serve as a suitable evaluation for treatment outcomes. The quantification of the severity of dizziness can represent the severity of treatment outcome by the results of vestibular tests, which can be applied especially in children (narrow ear canal) and in severe otitis media.^[14]^ Timely diagnosis and appropriate outcome assessment can effectively shorten hospitalization and prevent disease progression. This method is similar to that used for the recovery of acute labyrinthitis,^[15]^ which evaluates the degree of dizziness to infer the effectiveness of the treatment. Thus, we used vestibular tests, including sPSG, cVEMP, and vHIT, to provide an objective assessment of every component and subsystem of balance, allowing specific profiling of the patient. The results are more convincing than caloric testing, which is the most widely used vestibular test to identify the presence and side of peripheral vestibular hypofunction. Caloric testing is less suitable in patients with external auditory canal swelling and infection. Apart from potentially exacerbating patient discomfort, it may also worsen the condition of infection. In this case, the improvements in sPSG and cVEMP correlated with the clinical course, but vHIT did not show a remarkable correlation. This study is a preliminary approach in which patients diagnosed with NTM otomastoiditis can be evaluated in the clinical course using vestibular tests if they have symptoms of dizziness. The utilization of vestibular testing as an alternative approach for tracking the clinical progress of patients with chronic cervicofacial infections accompanied by vertigo, particularly when conventional otoscopy is challenging, could serve as a noteworthy consideration.

In conclusion, NTM is a rare cause of otomastoiditis. A high index of suspicion is required for diagnosis to avoid prolonged morbidity. In this severe case of NTM otomastoiditis combined with meningitis and dizziness, imaging examinations and quantification of the severity of dizziness should be highlighted. To our knowledge, this is the first case followed by vestibular tests for the treatment outcomes of antibiotic regimens in patients with NTM mastoiditis. We envision that this approach will be applicable for monitoring disease treatment progress in patients with chronic cervicofacial or otic infections accompanied by dizziness, extending beyond NTM mastoiditis. In the future, we plan to collect more clinical data and expect to provide a convenient and cost-effective method of assessment for such patients.

## Acknowledgments

We would like to thank Lee YJ for assistance with mycobacteriology culture and the revision of this case report.

## Author contributions

**Conceptualization:** Hann-Ziong Yueh, Che-Hsuan Lin.

**Data curation:** Hann-Ziong Yueh, Hung-Lun Chu, Shih-Chun Lu.

**Investigation:** Hann-Ziong Yueh, Hung-Lun Chu, Shih-Chun Lu.

**Methodology:** Hung-Lun Chu, Shih-Chun Lu.

**Project administration:** Hann-Ziong Yueh, Shih-Chun Lu.

**Supervision:** Yuarn-Jang Lee, Che-Hsuan Lin.

**Validation:** Hung-Lun Chu.

**Writing – original draft:** Hann-Ziong Yueh, Hung-Lun Chu.

**Writing – review & editing:** Che-Hsuan Lin.
